# How message appeals and prior product use influence information processing, risk perceptions, trust, attitudes, and genetic test purchase intentions

**DOI:** 10.1371/journal.pone.0283102

**Published:** 2023-03-15

**Authors:** Matthew S. VanDyke, Nicole M. Lee, Alan Abitbol, Stephen W. Rush

**Affiliations:** 1 Department of Advertising & Public Relations, The University of Alabama, Tuscaloosa, Alabama, United States of America; 2 School of Social & Behavioral Sciences, Arizona State University, Phoenix, Arizona, United States of America; 3 Department of Communication, University of Dayton, Dayton, Ohio, United States of America; 4 Mike Curb College of Entertainment & Music Business, Belmont University, Nashville, Tennessee, United States of America; St John’s University, UNITED STATES

## Abstract

Within the direct-to-consumer (DTC) genetic test industry, attracting customers can be difficult especially due to the highly sensitive nature of these products. How these tests are communicated to consumers may be one avenue in which companies can impact customer purchase intentions. A 2 (message sidedness: one-way vs. two-way refutational) x 2 (hedging: present vs. absent) between-subjects experiment was conducted to understand how message features and prior product use influence information processing, risk and trust perceptions, and attitude toward the genetic test, which in turn, may influence direct-to-consumer (DTC) genetic test purchase intentions. Results demonstrated that having used a genetic test in the past predicted participants’ trust in the company, information processing, and risk judgments; however, among those who used a genetic test, viewing a message that included hedging tended to increase their trust in the message. Trust in the message and company, information processing, and risk judgments significantly predicted participants’ attitudes toward genetic testing, which in turn predicted their purchase intentions. The results suggest that in the context of DTC genetic test messaging, practitioners should strive to increase consumer trust in the message and the company and facilitate information processing, and they should work to diminish perceived risk. These results suggest opportunities for identifying other message features that may influence message and company trust, information processing, risk judgments, and attitudes related to DTC genetic testing.

## Introduction

Since hitting the market in the early 2000s, direct-to-consumer (DTC) genetic tests such as those produced by 23andMe and Ancestry.com have become increasingly popular. As of July 2019, 15% of U.S. adults had taken one or more DTC tests to obtain health and/or ancestral information [1]. As the prevalence of such tests has grown, so have concerns about the ability of consumers to understand and act upon the information provided [2]. In particular, medical professionals have expressed concerns about the clinical utility of DTC genetic tests [3] and about consumers making health decisions based on their results without guidance from a physician or genetic counselor [4, 5]. Test results indicating average or below average risk for a disease or condition may give consumers a false sense of security because tests only include specific genetic variants and do not account for other risk factors [6]. On the other hand, results indicating increased risk may cause unnecessary worry in instances where absolute risk (as opposed to relative risk) is still very low or when there are no additional screenings or treatments available for the condition, meaning the consumer cannot act on the information they receive.

DTC genetic tests represent a unique communication context as companies are essentially selling a scientific product directly to consumers by collecting genetic samples and other sensitive information. Because of this, concerns have been raised regarding privacy and information security [7]. Thus, such companies need to balance traditional product promotion strategies with information communicating the science and risks associated with the product and services provided. Accordingly, this study was informed by the message sidedness literature—as message sidedness is a common variable used to compare the influence of promoting a position (e.g., “buy this genetic test!”) against promoting a position while also inoculating against an opposing position (e.g., “buy this genetic test, because it’s better than others!”)—and the hedging literature—to examine the impact of disclosing uncertainties and limitations inherent in DTC genetic test products. These variables afforded the opportunity to examine and compare the influence of traditional product promotion (message sidedness) and communication of scientific risk (hedging) in a DTC health product context. Previous research in this area analyzed social media content from 23andMe to understand how the popular DTC genetic testing company balances promotional and science content [8]. Still, scholars have called for more research examining communication strategies used in for-profit science communication [9] and there are opportunities to examine the processes and effects of how message strategies may influence consumers’ perceptions and purchase intentions related to DTC genetic testing.

Pharmaceutical advertising has dominated most scholarly attention in the DTC communication space related to science, health, and risk. Researchers analyzed content of DTC prescription drug commercials and websites [10–12], the presentation of risk information on branded drug websites [13], the presence of third-person effects among older consumers [14], how consumer reliance on certain information channels associates with attitudes toward DTC prescription drug advertising [15], the comparative effects of different advertising formats [16], and how DTC pharmaceutical advertising might impact patient-physician interactions [17, 18]. In these examples, the message is delivered directly to consumers but the products still require a physician’s prescription unlike DTC genetic testing, where consumers independently make purchase decisions. Scant research in this area has examined how message features promoting DTC health products may impact individuals’ perceptions and purchase intentions—a gap this current study aims to fill.

Because of the inherent risk and limitations endemic to DTC genetic testing products and services combined with the company’s need to promote its products and services, the current study relied on the message sidedness and hedging literatures to investigate the effects of a company acknowledging uncertainties and limitations associated with its genetic testing product—while simultaneously differentiating the product from its competitors—on product purchase intentions. Using theoretical linkages from the risk information seeking and processing model and the theory of planned behavior [19], the specific goal of this study was to examine how message sidedness—incorporating a two-sided refutational or one-sided persuasive message—and hedging—the inclusion or omission of language that acknowledges a product’s or service’s limitations, caveats, or uncertainties—interact with prior product use to influence perceptions and purchase intentions related to a DTC genetic test. A 2 (message sidedness: two-sided refutational vs. one-sided) x 2 (hedging: present vs. absent) online experiment was conducted to examine main and interaction effects with prior DTC genetic test use to examine the impacts of personal experience and message features on individuals’ trust of the message and company, information processing, risk judgment, and attitude toward the DTC genetic test. In turn, the impacts of message and company trust, information processing, risk judgment, and attitude toward the genetic test on DTC genetic test purchase intentions were examined. Results hold theoretical and practical implications regarding the role of message appeals and individual differences in predicting DTC genetic test purchase intentions.

## Literature review

### The Theory of Planned Behavior and Risk Information Seeking and Processing Model

Theory of Planned Behavior is a psychological theory through which research findings have offered heuristic value in demonstrating the influence of attitudes, subjective norms, and perceived behavioral control in influencing an individual’s behavioral intentions [20, 21]. Similarly, research guided by the Risk Information Seeking and Processing Model has afforded heuristic value in demonstrating that individuals’ processing of risk information depends on their predispositions and experiences, evaluations of risk and perceived institutional trust, levels of concern, and perceived information need [22, 23]. Prior research has combined theoretical linkages from the theory of planned behavior and the risk information seeking and processing model to understand how factors from both frameworks—including risk information processing behaviors, perceived hazard characteristics (including risk judgments and institutional trust), subjective norms, and attitude toward an action (e.g., purchasing a product)—influenced individuals’ risk decision-making (e.g., participating in a clinical trial) [19]. The results demonstrated, in part, that information processing, risk judgments, and institutional trust were antecedent to attitudes, and attitudes significantly predicted participants’ behavioral intentions.

Given the important roles of attitudes, risk judgments, information processing, and trust in predicting behavioral intentions as documented by previous literature, these variables were examined in the current study. Similarly, given the observation that much of this research adopted a cross-sectional survey approach, our aim was to conduct an experiment investigating whether selected theory of planned behavior and risk information seeking and processing constructs explained processes between message appeals in a DTC health product context and individuals’ intention to purchase a DTC genetic test. Specifically, we examined how trust in the message and company, risk information processing, risk judgment, and belief-based attitudes mediated the effects of message appeals and prior product experience on individuals’ intentions to purchase a DTC genetic test.

### Risk information processing, risk judgment, and trust

Research demonstrates that individuals often vary in the extent to which they exert energy toward processing information based on their motivation and/or ability to process information [24]. When considering whether or not to purchase a DTC health product, such as a genetic test, it is likely important for prospective buyers to consider the product’s potential risks and benefits [25], relying on a more effortful mode of information processing to make a purchase decision; systematic processing has been demonstrated as a predictor of belief-based attitudes [26], and the theory of planned behavior proposes that individuals typically consider readily accessible beliefs about possible behavioral consequences, which tend to predict an individual’s behavior [27].

In the context of DTC genetic testing, risk concerns often relate to privacy and information security [7]. Risk perceptions constitute individuals’ estimations of the likelihood and potential severity of risks [28]. The way product information is presented to consumers can affect cognitive and affective evaluations of the product [29, 30], and research has shown that risk judgments (e.g., of privacy risk) can be strong antecedents of consumer behavior [31]. For example, perceived benefits (e.g., price and convenience) and trust positively predicted consumers’ attitudes toward online shopping [32], and trust and risk perceptions have been shown to predict positive organizational outcomes, such as customer satisfaction, return visits, and positive word-of-mouth [33]. Similarly, research demonstrated that trust is a positive predictor of attitudes and purchase intentions [34]. Consumers’ risk perceptions have been shown to mediate the influence of messaging on attitude toward health-related advertising [35], and risk perceptions have been found to negatively relate to positive attitudes [19]; however, greater trust and engaging in systematic processing tended to yield more favorable attitudes toward behavior and greater behavioral intentions [19]. Research also has demonstrated that corporate trustworthiness and attitude toward an advertiser predicted attitudes toward biotechnology and purchase intentions [36].

Taken together, prior research supports the important roles of information processing, organization and message trust, and risk judgments in predicting consumer attitudes and purchase intentions. Informed by the literature, the following hypotheses were proposed:

H1: Participants’ (a) trust in the message, (b) trust in the company, and (c) information processing will positively predict their attitude toward genetic testing. Participants’ (d) risk judgment will negatively predict their attitude toward genetic testing.

H2: Attitude toward genetic testing will positively predict participants’ genetic test purchase intention.

### Prior product use

Because prior experience may also predict future behaviors, and because consumers can take more than one type of test or engage other services offered by DTC genetic testing companies, prior product use may be a predictor of future product use. Consumers with prior brand experience may more easily recall the brand and pay more attention to it [37], and prior experience may have a stronger direct effect on consumer behavior than potential intervening variables, such as trust and perceived risk [31]. Indeed, when considering antecedents to product purchase intentions, previous purchasing behaviors and consumer experiences tend to predict their subsequent purchase intentions and behaviors [38, 39]. Related, previous experience with risk is an established predictor of risk judgments [40], perhaps due to the role of affect and availability heuristics afforded by previous experiences, which make risk judgments more salient [41]. Brand familiarity and experience have been shown to influence risk perceptions and purchase intentions [42, 43]. The risk information seeking and processing model predicts that previous risk experiences are antecedent to risk judgments, institutional trust, and information processing among other variables [44], effects that have endured over years of testing the model [45].

Taken together, previous research suggests that prior use of a genetic test product should predict individuals’ trust of a DTC genetic testing company and message, should motivate information processing due to past experience with the product, and should predict related risk perceptions and attitudes toward the genetic test. Thus, the following hypothesis was proposed:

H3: Prior use of a genetic test will positively predict (a) trust in the message, (b) trust in the company, (c) information processing, (d) risk judgment, and (e) attitude toward genetic testing.

### Message appeals

Research in marketing, management, and consumer behavior has demonstrated the impact that various message characteristics (e.g., prevention- versus promotion-focused messages; pro-environmental appeals) may have on consumer decision-making and purchase intentions [46–48]. The current study aimed to advance this literature by understanding how two message appeal variables—message sidedness and hedging—might affect consumers’ attitude toward purchasing a genetic test and other potential mediating and moderating individual differences.

#### Message sidedness

Businesses often compete to increase their share of consumers buying their products or services [49]. In such cases, businesses have the option to inoculate against their competitors’ arguments or to completely ignore competitors’ opposing arguments [50]. These options are explicated in the message sidedness literature and can be further distinguished as one- or two-sided messages. A one-sided message simply bolsters one side of an argument while ignoring the other. A two-sided message discusses an argument from both points of view (e.g., the supporting and opposing points of a perspective). Two-sided messages may be further distinguished as refutational two-sided messages and non-refutational two-sided messages [50]. A refutational two-sided message supports the primary argument while counter-arguing the opposition. However, a non-refutational two-sided message supports the primary argument and acknowledges the opposition points but makes no attempt to refute opposing viewpoints [50].

Message sidedness research has been conducted often in advertising contexts. While some early research revealed no significance when examining message sidedness effects [51, 52], refutational two-sided messages typically are found to be more persuasive than one-sided messages, which tend to be more persuasive than non-refutational two-sided messages [53–55]. However, non-refutational two-sided messages appear to have different effects in consumer advertising contexts. In advertisements, non-refutational two-sided messages are neither more nor less persuasive than one-sided advertisements [50]. That is, non-refutational two-sided advertising messages do not seem to suffer the same negative persuasion consequences that parallel non-advertising messages do. Because of the lack of differences between non-refutational two-sided messages and one-sided messages in advertising contexts, and the practical reality that businesses likely would not pay for advertising copy to present supporting and opposing viewpoints without refuting the latter, the current study only manipulated message sidedness as either a two-sided refutational message or one-sided message.

Researchers have found that refutational two-sided messages tend to generate more supportive thoughts and less counterarguing than one-sided messages [56]. Moreover, research examining message sidedness in science content revealed that participants were more likely to choose two-sided articles, to read them first, and to read them longer than one-sided messages; participants with a high need for cognition were found to favor two-sided messages [57]. These findings suggest that two-sided refutational messages should perhaps increase trust in the message and company and favorable attitudes toward a genetic test, facilitate information processing, and perhaps decrease risk perceptions. These predictive effects should hold particularly when participants have used a DTC genetic test in the past. Thus, the following hypotheses were proposed:

H4: Two-sided refutational messages will be more persuasive than one-sided messages in affecting (a) trust in the message, (b) trust in the company, (c) information processing, and (d) attitude toward genetic testing, and (e) two-sided messages will decrease risk judgments more than one-sided messages.

H5: Message sidedness and prior product use will interact such that among participants who have used a genetic test, two-sided refutational messages will positively predict (a) trust in the message, (b) trust in the company, (c) information processing, (d) risk judgment, and (e) attitude toward genetic testing.

#### Hedging

Science often contributes to advertising copy in more or less explicit ways. For example, the inclusion of research results in advertising (e.g., to validate product performance claims) has long been practiced, and scholarship has examined consumer believability of such information [58]. One characteristic of science is that it is tentative [59], and communicating about science-related topics necessitates consideration of communicating about uncertainties related to the extent to which something is known and the degree of confidence surrounding it [60].

Hedging is defined as the use of language to express tentativeness or caution when communicating about information [61]. In a scientific context, hedging is language that contains caveats, limitations, or other indicators of scientific uncertainty [60]. Hyland [62] suggested that hedging can be lexical (e.g., manifested in single words or phrases, such as *could* or *might*) or it can be discourse-based (e.g., manifested in entire sentences describing the limitations of research). Jensen [60] suggested that hedging may be thought of as a form of powerless language because it is relatively uncertain and qualified compared to more powerful language that is both certain and assertive; presumably more powerful language is common in for-profit advertising contexts, but perhaps there are advantages to incorporating hedging into company messaging related to consumer trust, information processing, risk judgments, attitudes, and in turn, purchase intentions.

Most communication research examining hedging effects focused on the role of hedging in news coverage, and the findings are somewhat mixed. For example, scientists and journalists were perceived as more trustworthy when news coverage of cancer research included information about study limitations and when such hedging was attributed to the scientists responsible for the research [60]. In the context of cancer research news coverage, research findings demonstrated that reading hedged news articles may decrease reported fatalism and proneness to nutritional backlash [63]. Research has shown that journalists may be perceived as more credible when their articles fully explain uncertainty related to research covered in news reports and include uncertainty disclosed by the scientist responsible for the covered study [64]. Other research has demonstrated that media coverage communicating scientific uncertainties may not affect beliefs, credibility perceptions, or behavioral perceptions [65], and highlighting uncertainties related to nanotechnologies had no effect on trust in scientists [66]. However, what are the implications of including hedged language in advertising?

Fuertes-Olivera and colleagues [67] argued that hedging is a meta-discourse strategy used by advertising copywriters and suggested that a foundational role of hedging in advertising is “to make indirect reference to the qualities of the goods being advertised…which shows the degree of tentativeness, possibility and/or politeness copywriters use in the messages” (p. 1299), allowing copywriters to “compose slogans and headlines with both appropriate caution and deference” (p. 1300). In this way, they argue that hedging in advertisements:

reinforce the truth value of the proposition, intensifying the addresser’s assertion that the slogans and/or headlines are true…tone down the message by lowering the force of the verb or predication…introduce some degree of doubt with regard to the truth value of slogans and headlines…[and] assure addressees do not intend to infringe on their freedom to act. (Fuertes-Olivera et al., 2001, 1300–1301)

So while the use of hedging may soften persuasive strategies, is this effect beneficial for companies or consumers?

Prior literature suggests an emphasis on disclosure and accuracy in company messaging should favorably influence consumer attitudes and trust [68]. Moreover, although disclosure of flaws has been demonstrated to negatively affect perceived expertise of a scientist, the same data demonstrated that disclosure can positively influence perceptions of integrity and benevolence [69].

Recent research in communication has examined the effects of sponsor disclosures on consumer trust and attitudes [70], which may also provide guidance. Although sponsorship disclosure and the use of hedging are categorically different, they both may be conceived as attempts at organizational transparency and, thus, may hold important implications for the current research. The disclosure of sponsored social media posts from influencers has been shown to negatively affect brand attitudes and influencer credibility; however, the negative effect only held when an influencer used a one-sided versus a two-sided message [71]. The research findings suggest that genuine, transparent communication efforts articulated through messaging, though, may be beneficial for the responsible brand. In line with these findings, the following hypotheses were proposed:

H6: The inclusion of hedging will positively affect (a) trust in the message, (b) trust in the company, (c) information processing, and (d) attitude toward genetic testing, and (e) negatively affect risk judgment.

H7: Hedging and prior product use will interact such that among participants who have used a genetic test, the inclusion of hedging will positively affect (a) trust in the message, (b) trust in the company, (c) information processing, and (d) attitude toward genetic testing, and (e) negatively affect risk judgment.

H8: Hedging and message sidedness will interact such that two-sided refutational messages that include hedging will be more persuasive than one-sided messages without hedging in affecting (a) trust in the message, (b) trust in the company, (c) information processing, and (d) attitude toward genetic testing, and (e) two-sided messages with hedging will decrease risk judgments more than one-sided messages without hedging.

## Method

### Design

This study employed a 2 (message sidedness: one-sided vs. two-sided refutational) x 2 (hedging: present vs. absent) between-subjects experimental design. The study was programmed, distributed, and data were collected via Qualtrics. Research participants were recruited and compensated through Qualtrics Online Panels.

### Procedure

This study was approved by The University of Alabama’s Institutional Review Board. Prospective participants were U.S. adults recruited from Qualtrics, a survey panel provider and survey software company. Participant recruitment and data collection occurred between September 3 and September 18, 2020. Individual participation occurred online via each participant’s personal electronic device. Recruited participants viewed an information page explaining the purpose of the study, participant rights, risk and benefits involved with participation, and contact information for the primary investigator and the investigator’s institutional review board. Respondents were notified via written text that advancing through the Qualtrics interface to complete the study demonstrated their consent to participate in the study. All participants who elected to participate in the study read the following statement upon advancing from the information page:

Direct-to-consumer genetic testing consists of genetic tests that are marketed directly to customers. Using these tests, customers send the company a DNA sample and receive results about their genetic information without necessarily involving a healthcare provider or health insurance company in the process.

You are about to see an advertisement for a genetic testing company. You will be asked about your opinions of the advertisement’s content, so please read the entire advertisement before advancing through the study.

After reading this statement, participants were randomly assigned to one of four conditions in which they viewed a one-sided message, a one-sided message with hedging, a two-sided refutational message, or a two-sided refutational message with hedging. Upon examining a print advertisement, participants answered manipulation check items, followed by items evaluating their trust in the message, information processing, risk judgment, trust in the company, belief-based attitudes, purchase intentions, and demographics.

### Measures

*Trust in the message* was measured using 20 items adapted from Soh, Reid, and King [72], including items assessing the degree to which information conveyed in the message was honest, truthful, credible, reliable, dependable, accurate, factual, complete, clear, valuable, good, useful, likable, enjoyable, positive, and helps people make the best decisions. Other items assessed participants’ willingness to rely on information from the message when making purchase decisions, and willingness to recommend the advertised product to friends and family. Responses to each item varied on a seven-point scale from strongly disagree (1) to strongly agree (7), and participants’ aggregate score from these items constituted their reported trust in the advertisement (α = .98; *M* = 5.10, *SD* = 1.17).

*Information processing* was measured using three items adapted from Johnson [73], including “while reading the ad, I thought about what actions I myself might take based on what I read,” “I found myself making connections between the information in the ad and what I have read or heard about elsewhere,” and “I thought about how what I read in the ad related to other things I know.” Responses to each item varied on a seven-point scale from strongly disagree (1) to strongly agree (7), and participants’ aggregate score from these items constituted the extent to which they processed information from the advertisement they read (α = .87; *M* = 5.01, *SD* = 1.37).

*Risk judgment* was measured using two items adapted from Yang and colleagues [19] that read “based on the advertisement you read, do you think that using a genetic test from this company could put you or your personal information at risk?” and “if using a genetic test from this company were to put you or your personal information at risk, how serious do you think the risk would be?” Responses to the items varied on seven-point scales from not at all (1) to very much (7) and from not at all serious (1) to very serious (7), respectively. Participants’ aggregate score from these items constituted their reported risk judgment (α = .82; *M* = 4.70, *SD* = 1.56).

*Company trust* was measured using nine items adapted from Cho, Huh, and Faber [74]. Responses to each item varied on a seven-point scale from strongly disagree (1) to strongly agree (7), and statements assessed the extent to which the participant believed the advertiser would provide the best genetic test on the market, would do its best to help the participant, would be honest regarding services and fees, would keep its commitments, is interested in providing quality services, is truthful, sincere and genuine, is capable of giving accurate and timely information, and is knowledgeable about issues related to genetic testing. Participants’ aggregate score from these items constituted their trust in the advertiser (α = .96; *M* = 5.08, *SD* = 1.13).

Participants’ *belief-based attitudes* toward the DTC genetic test were measured using seven items adapted from Lafferty and Emondson [75] and Yang and colleagues [19]. Responses to three items varied on a seven-point scale from strongly disagree (1) to strongly agree (7) and assessed participants’ beliefs about whether purchasing a genetic test from the company would help them understand more about themselves, their relatives and their health. Four semantic differential items assessed whether the company’s genetic tests were perceived as bad/good, unfavorable/favorable, negative/positive, not worth the effort/worth the effort. Participants’ aggregate score from these items constituted their belief-based attitude (α = .94; *M* = 5.33, *SD* = 1.26).

*Purchase intention* was measured using three items adapted from Lafferty and Emondson [75] assessing how likely participants would be to purchase a genetic testing kit from the advertiser. Responses ranged from not very likely (1) to very likely (7), definitely would not consider it (1) to definitely would consider it (7), and not at all probable (1) to very probable (7). Participants’ aggregate score from these items constituted their reported purchase intention (α = .95; *M* = 4.64, *SD* = 1.83).

There were no more than three missing data points per item. Each missing value was replaced with the respective series mean.

### Stimuli

The stimuli for this experiment consisted of four manipulated print advertisements designed to appear produced by fictional DTC genetic test company Xistance. Each advertisement featured a Caucasian female smiling and carrying a backpack on the right side of the ad, and featured ad copy on the left side. Each advertisement encouraged the reader to “Use your DNA to explore your unique story!”, listed three product benefits, including “Learn more about your genetic traits,” Connect with your genetic relatives” and “Discover your genetic risk for disease” with text expounding on each benefit below each point, and closed with a call to action: “Visit xistance.com to get started today!”. All advertisement elements remained constant between conditions with the exception of ad copy expounding on each of the three product benefits. One-sided messages included one sentence detailing each product benefit. Two-sided messages added an additional sentence per listed product benefit to differentiate Xistance from its competitors. The hedging manipulation added a clause to the one-sided message manipulation acknowledging caveats, limitations, or uncertainties related to the listed product benefit. The stimuli are provided in the S1 Appendix.

Stimuli were pre-tested prior to the experiment to ensure the intended manipulations manifested. Pre-test participants (*N* = 40) were primarily female (*n* = 26; 65%), white (*n* = 29; 72.5%; Black or African American: *n* = 8; 20%; Asian: *n* = 2; 5%), and did not identify as Hispanic/Latino (*n* = 34; 85%). The average age of pre-test participants was 33.50 (*SD* = 9.82) and most participants (*n* = 33; 82.5%) reported having not used a direct-to-consumer genetic testing product in the past. As the results in Table 1 demonstrate, two-sided messages were perceived to acknowledge two perspectives compared to one-sided ads. Two-sided ads were perceived as more refutational than one-sided messages, though the one-sided hedging advertisement was perceived as more refutational than the one-sided ad without hedging. Similarly, advertisements that included hedging were perceived to highlight limitations and uncertainties more so than ads that excluded hedging. No differences were observed between ad conditions in terms of their perceived advocacy, their comprehensibility, or perceived interest.

**Table 1:** Stimuli pre-test results.

Measure	One-sided/No Hedging	One-sided/Hedging	Two-sided/No Hedging	Two-sided/Hedging
	*M* (*SD*)	*M* (*SD*)	*M* (*SD*)	*M* (*SD*)
Advocate	5.05 (1.68)^A^	4.80 (1.65)^A^	5.10 (1.46)^A^	4.60 (1.61)^A^
Both Positions	3.43 (2.17)^A^	3.93 (1.89)^A^	4.82 (1.96)^B^	5.08 (1.67)^B^
Refute	3.35 (2.03)^A^	3.95 (1.95)^B^	4.72 (1.89)^C^	4.87 (1.65)^C^
Hedging	3.96 (1.97)^A^	5.35 (1.34)^B^	4.41 (1.57)^A^	5.04 (1.45)^B^
Comprehension	5.63 (1.23)^A^	5.48 (1.18)^A^	5.58 (1.17)^A^	5.41 (1.29)^A^
Interest	4.96 (1.48)^A^	5.11 (1.36)^A^	5.17 (1.32)^A^	5.09 (1.24)^A^

Note: All means not sharing a superscript are different by Bonferroni post-hoc test horizontally.

### Respondents

The majority of participants (*N* = 203) was female (*n* = 116, 57.14%) and white (*n* = 154, 75.86%). Black or African Americans (*n* = 23) made up 11.33%, followed by Asian (*n* = 20, 9.85%) then American Indian or Alaska Native (*n* = 2, 0.99%). The majority did not identify as Hispanic/Latino (*n* = 189, 93.10%). The average age of participants was 42.04 (*SD* = 15.22) and most participants (*n* = 143; 70.44%) reported having not used a direct-to-consumer genetic testing product in the past. Most participants identified as Democrat (*n* = 73, 35.96%), followed by Republican (*n* = 68, 33.50%), Independent (*n* = 60, 29.56%), and another political affiliation (*n* = 2, 0.99%). Regarding political ideology, the sample leaned slightly liberal (*M* = 3.93, *SD* = 1.93). The majority of participants held a four-year degree (*n* = 60, 29.56%), followed by those who had completed some college (*n* = 40, 19.70%), who held a graduate degree (*n* = 37, 18.23%), high school graduates (*n* = 29, 14.29%), those who held a two-year degree (*n* = 18, 8.87%), held a professional degree (*n* = 10, 4.93%), held a doctorate (*n* = 8, 3.94%), and those with less than a high school education (*n* = 1, 0.49%).

## Results

A manipulation check was conducted as part of the main experiment. The results demonstrated that one-sided messages were perceived as more one-sided (*M* = 4.75, *SD* = 1.60) than two-sided (*M* = 4.31, *SD* = 1.68), *t*(201) = 1.88, *p* = .06. Similarly, two-sided messages were perceived as more two-sided (*M* = 5.09, *SD* = 1.46) than one-sided (*M* = 4.30, *SD* = 1.99), *t*(201) = 3.20, *p* = .002; two-sided messages (*M* = 4.37, *SD* = 1.51) were also perceived as more refutational than one-sided messages (*M* = 3.60, *SD* = 1.83), *t*(201) = 3.25, *p* = .001. Hedging messages (*M* = 4.95, *SD* = 1.20) were perceived to highlight limitations and uncertainties more so than messages that contained no hedging (*M* = 4.29, *SD* = 1.70), *t*(201) = 3.20, *p* = .002. As the results indicate, the manipulations were successful.

**Fig 1:**
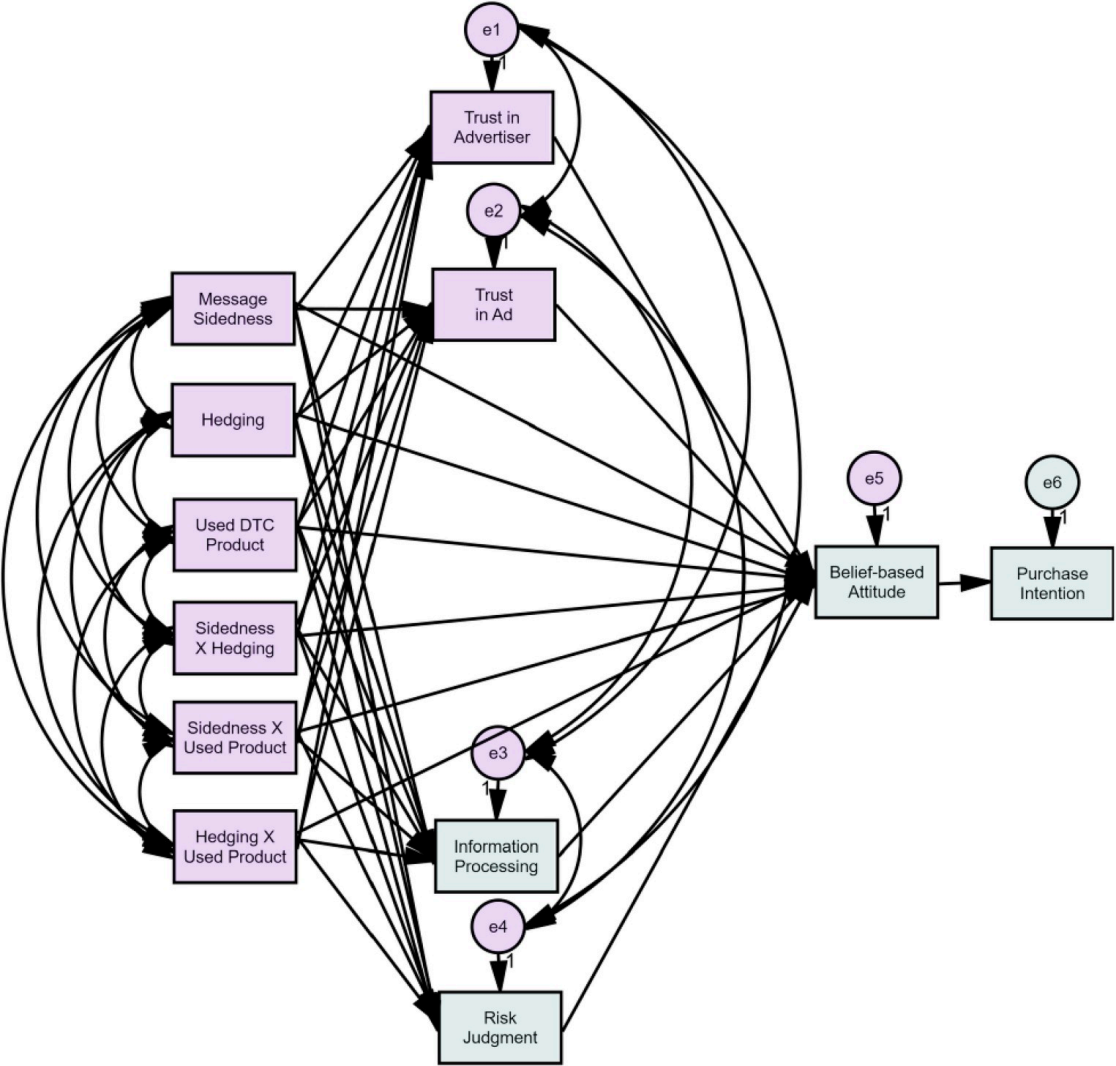
Hypothesized path model.

**Fig 2:**
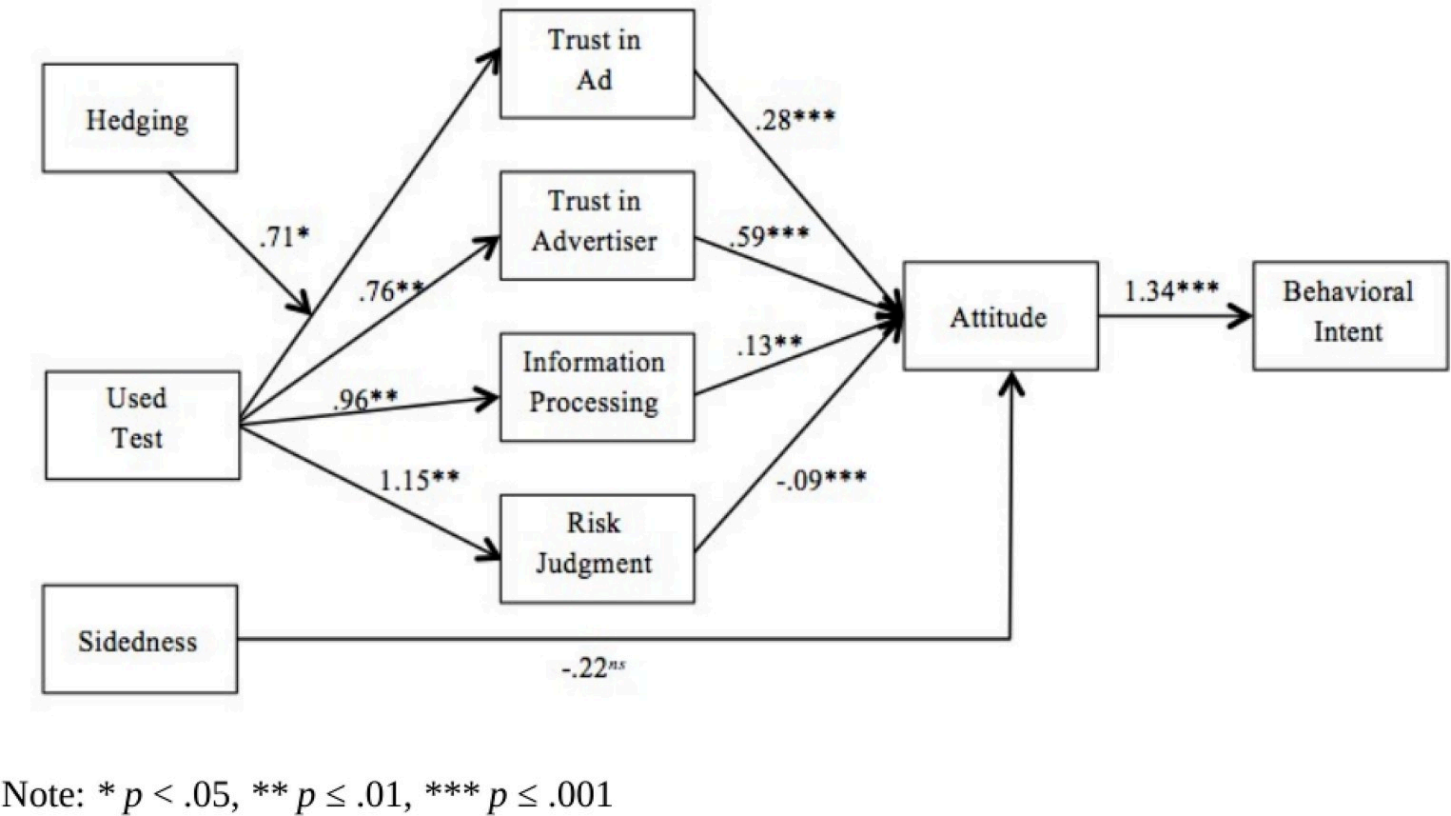
Analyzed path model omitting most non-significant paths to reduce visual clutter.

To test the hypotheses, a structural equation model was calculated using AMOS 25. Fig 1 shows the model that was analyzed. Significance tests for direct, indirect, and total effects were estimated using bootstrapping procedures employing 5,000 bootstrap samples with a 95% bias-corrected confidence interval. Analysis of the initial structural equation model revealed an adequate fit, (*χ2*(10) = 30.35, *p* = .001, *χ2*/df = 3.03, CFI = .99, RMSEA = .10, AIC = 166.35). However, inspection of the modification indices suggested that model fit could be improved if the error terms of attitude and behavioral intention were allowed to correlate. The fit of the modified model was good upon adding the correlated error terms (*χ2*(9) = 5.58, *p* = .78, *χ2*/df = .62, CFI = 1.00, RMSEA = .00, AIC = 143.58). Fig 2 shows the analyzed path model, omitting non-significant paths to reduce visual clutter. Table 2 shows the path analysis model estimates, and Tables 3 and 4 show the covariance and correlations matrices.

**Table 2:** Final estimates of the path analysis model.

Path		*B*	SE	*p*-value
Message Sidedness → Trust in Ad		.15	.24	.54
Message Sidedness → Trust in Advertiser		.19	.23	.42
Message Sidedness → Information Processing		-.04	.28	.87
Message Sidedness → Risk Judgment		.14	.33	.68
Message Sidedness → Attitude		-.22	.13	.10
Hedging → Trust in Ad		.03	.23	.89
Hedging → Trust in Advertiser		.19	.22	.40
Hedging → Information Processing		-.26	.27	.32
Hedging → Risk Judgment		.02	.32	.94
Hedging → Attitude		-.07	.13	.56
Used Product → Trust in Ad		.48	.29	.11
Used Product → Trust in Advertiser		.76	.28	.006
Used Product → Information Processing		.96	.34	.004
Used Product → Risk Judgment		1.15	.40	.004
Used Product → Attitude		.04	.17	.83
Sidedness X Hedging → Trust in Ad		-.49	.31	.11
Sidedness X Hedging → Trust in Advertiser		-.47	.30	.11
Sidedness X Hedging → Information Processing		-.13	.36	.71
Sidedness X Hedging → Risk Judgment		-.35	.42	.42
Sidedness X Hedging → Attitude		.16	.17	.35
Used Product X Sidedness → Trust in Ad		-.02	.34	.95
Used Product X Sidedness → Trust in Advertiser		-.07	.33	.84
Used Product X Sidedness → Information Processing		-.13	.39	.74
Used Product X Sidedness → Risk Judgment		-.19	.47	.69
Used Product X Sidedness → Attitude		.02	.18	.91
Used Product X Hedging → Trust in Ad		.71	.34	.04
Used Product X Hedging → Trust in Advertiser		.41	.32	.21
Used Product X Hedging → Information Processing		.57	.39	.15
Used Product X Hedging → Risk Judgment		-.37	.47	.43
Used Product X Hedging → Attitude		-.03	.19	.86
Trust in Ad → Attitude		.28	.07	< .001
Trust in Advertiser → Attitude		.59	.08	< .001
Information Processing → Attitude		.13	.05	.005
Risk Judgment → Attitude		-.09	.03	.001
Attitude → Purchase Intention		1.34	.07	< .001

**Table 3:** Covariance matrix.

	Used Product X Hedging	Used Product X Sidedness	Used Product	Sidedness X Hedging	Hedging	Message Sidedness	Risk Judgment	Information Processing	Trust in Ad	Trust in Advertiser	Attitude	Intent
Used Product X Hedging	.122											
Used Product X Sidedness	.065	.139										
Used Product	.101	.118	.208									
Sidedness X Hedging	.053	.047	.014	.188								
Hedging	.070	.004	-.007	.124	.250							
Message Sidedness	.018	.084	.020	.126	-.001	.250						
Risk Judgment	.045	.082	.178	-.056	-.072	-.008	2.430					
Information Processing	.131	.121	.241	-.026	-.050	-.009	.582	1.873				
Trust in Ad	.112	.089	.163	-.026	-.006	-.005	.274	1.053	1.361			
Trust in Advertiser	.114	.102	.188	-.012	.012	.005	.235	1.062	1.119	1.275		
Attitude	.112	.086	.176	-.020	.004	-.032	.073	1.114	1.151	1.180	1.569	
Intent	.149	.116	.236	-.027	.006	-.043	.098	1.492	1.541	1.581	1.817	3.337

**Table 4:** Correlation matrix.

	Used Product X Hedging	Used Product X Sidedness	Used Product	Sidedness X Hedging	Hedging	Message Sidedness	Risk Judgment	Information Processing	Trust in Ad	Trust in Advertiser	Attitude	Intent
Used Product X Hedging	1.000											
Used Product X Sidedness	.495	1.000										
Used Product	.630	.692	1.000									
Sidedness X Hedging	.348	.288	.073	1.000								
Hedging	.402	.020	-.031	.571	1.000							
Message Sidedness	.101	.451	.090	.582	-.005	1.000						
Risk Judgment	.082	.140	.251	-.083	-.092	-.010	1.000					
Information Processing	.274	.237	.386	-.043	-.073	-.013	.273	1.000				
Trust in Ad	.274	.204	.307	-.052	-.011	-.008	.151	.660	1.000			
Trust in Advertiser	.290	.242	.365	-.024	.021	.008	.134	.687	.850	1.000		
Attitude	.255	.185	.308	-.037	.007	-.051	.037	.650	.788	.834	1.000	
Intent	.234	.170	.283	-.034	.006	-.047	.034	.597	.723	.766	.794	1.000

Hypotheses 1a-1d predicted positive effects of participants’ trust in the company, trust in the message, and information processing on their attitude toward the genetic test, and a negative effect of risk judgment on attitude toward the genetic test. Analysis revealed that trust in the message (*b* = .28, *p* < .001), trust in the company (*b* = .59, *p* < .001), and information processing (*b* = .13, *p* = .005) positively predicted participants’ attitude toward the genetic test. Participants’ reported risk judgment negatively predicted their attitude toward the genetic test (*b* = -.09, *p* = .001). Thus, hypotheses 1a-1d were supported.

The second hypothesis predicted a positive effect of attitude toward the genetic test on participants’ genetic test purchase intention. As Table 2 demonstrates, attitude toward the genetic test positively predicted participants’ reported purchase intention (*b* = 1.34, *p* < .001), supporting the second hypothesis.

Hypotheses 3a-3e predicted that prior use of a genetic test would positively predict participants’ trust in the company and message, information processing, risk judgment, and attitude toward the genetic test. As Table 2 demonstrates, prior use of a genetic test positively predicted participants’ trust in the company (*b* = .76, *p* = .006), their information processing of advertising content (*b* = .96, *p* = .004), and their risk judgment (*b* = 1.15, *p* = .004). However, prior product use did not directly impact participants’ trust in the message or their attitudes toward the genetic test. Thus, H3a, H3c, and H3d were supported; H3b and H3e were not supported.

Hypotheses 4a-4e predicted the influence of message sidedness on participants’ trust in the company and message, information processing, risk judgment, and attitude toward the genetic test. Results revealed no significant relationship between message sidedness or any dependent variables; therefore, these hypotheses were not supported.

Hypotheses5a-5e predicted the interaction effects of message sidedness and prior product use on participants’ trust in the company and message, information processing, risk judgment, and attitude toward the genetic test. As Table 2 demonstrates, no significant interaction effects emerged, and these hypotheses were not supported.

Hypotheses 6a-6e predicted the impacts of hedging on participants’ trust in the company and message, information processing, risk judgment, and attitude toward the genetic test. Results demonstrated that hedging did not have a direct effect on any of the dependent variables; therefore, these hypotheses were not supported.

Hypotheses 7a-7e predicted the interaction effects of hedging and prior product use on participants’ trust in the company and message, information processing, risk judgment, and attitude toward the genetic test. As the results indicate, the only significant interaction effect that emerged was the effect on trust in the message (*b* = .71, *p* = .04). That is, among those who used a genetic test prior, the inclusion of hedging tended to increase their perceived trust in the message. Hypothesis 7a was supported; hypotheses 7b, 7c, 7d, and 7e were not supported.

Hypotheses 8a-8e predicted interaction effects between message sidedness and hedging on participants’ trust in the company and message, information processing, risk judgment, and attitude toward the genetic test. Results revealed no significant interaction effects; thus, these hypotheses were not supported.

Overall, the results indicated that prior use of a genetic test was an important antecedent to predicting individuals’ trust in a DTC genetic test company, their information processing of message content, and their risk judgment. However, prior product use did not directly impact individuals’ trust in the message or their attitudes toward the genetic test. Message appeals did not have a significant influence on individual difference variables, with the exception of a significant interaction effect between hedging and prior product use on trust in the message. Among those who used a genetic test prior to the experiment, the inclusion of hedging in a message tended to increase their perceived trust in the message. Trust in the message, trust in the company, and information processing positively predicted individual attitudes toward the genetic test, while risk judgments negatively predicted attitudes toward the genetic test. Moreover, attitude toward the genetic test positively predicted individual purchase intentions.

## Discussion

This study aimed to understand how message sidedness and hedging interact with prior product use to influence consumer trust, risk perceptions, information processing, attitudes, and purchase intentions related to DTC genetic tests. The results demonstrated that having used a genetic test in the past positively predicted participants’ trust in the company, greater information processing, and greater risk judgments; however, among those who previously used a genetic test, the inclusion of hedging in a promotional message tended to increase their trust in the message. These findings are not surprising as prior experience—and presumably positive experiences with a DTC genetic test provider—likely contributed to the direct effect on company trust. Similarly, participants who had taken a test likely had greater understanding of such products and services and likely compared their experiences and knowledge to what was being advertised. Because of this greater knowledge, participants who had taken a test likely were more familiar with both the benefits and risks of engaging such products, thus promoting greater realization of risk susceptibility and severity associated with engaging genetic testing products and services. However, contrary to prior research, prior product use did not directly impact attitudes toward the genetic test. Trust in the company and message, information processing, and risk judgment significantly predicted participants’ attitudes toward the genetic test, which in turn predicted their intentions to purchase a DTC genetic test. Sidedness and hedging message appeals did not affect any outcomes except that among those who had previously used a genetic test, the inclusion of hedging tended to increase their trust in the message. This finding suggests that when companies attempt to engage existing customers, messaging that includes tentative or cautious language about their products and services will be perceived as more trustworthy, which perhaps may yield positives outcomes for brand affinity, brand loyalty, and organization-public relationships—explorations for future research. These results suggest researchers should work to better understand other message appeals that may influence message and company trust, information processing, risk judgments, and belief-based attitudes; perhaps different message features are consequential in affecting such variables, which would hold important implications for DTC health product promotion and health risk communication theory and practice.

Theoretically, although these findings support prior theory of planned behavior and risk information seeking and processing evidence that trust, risk perceptions, and information processing are consequential for attitudes, which are in turn consequential for behavior, the current study expands our understanding of these relationships into a DTC health product context. That is, this study provides evidence for how message appeals and individual difference variables may operate in direct-to-consumer health settings in which companies (without mediation from a healthcare provider) communicate directly with consumers—a relatively new context that has received scant attention to date. Future research should continue to advance our understanding for how company-consumer interactions might impact consumer (health) risk decision-making compared to more traditional health/risk interactions (e.g., between a healthcare provider and patient). The current results suggest that specific to the context of this study, researchers should test other potential message features that may influence message and company trust, information processing, risk judgments, and attitudes related to DTC genetic tests to understand how message design choices in this domain may influence important outcomes—particularly given that these variables appear consequential in determining attitudes toward genetic tests and purchase intentions. Similarly, the current study examined risk perceptions specific to privacy and data security; however, future studies may incorporate other consumer concerns such as discovering unwanted information or not knowing how to act on the discovered information, which would inform theory and practice in this context. Finally, this study presents opportunities to study these variables in other DTC health contexts such as at-home COVID-19 tests, at-home allergy testing, or EKG monitors linked to smart phones. Future research should test and compare message strategies used to promote different types of DTC health products and services. Different message strategies in these domains may differentially impact consumer variables of interest and theoretically and practically meaningful message features will likely vary by context. For example, as the hedging literature suggests, perhaps manipulating powerful, certain, assertive language (e.g., “You must take this test!”) versus uncertain, qualified language (e.g., “The test typically is more effective than competitors”) in company promotional materials would yield important findings; future research also should explore the ethics of choosing certain message appeals over others.

The present study’s findings demonstrate that in the context of DTC genetic test advertising, practitioners should strive to build consumer trust in the message, the company, and work to facilitate information processing and diminish perceived risks. Trust in the message and company and information processing positively predicted belief-based attitudes about the genetic test, and risk judgments negatively predicted attitudes; similarly, attitudes positively predicted purchase intentions. The results offer nuanced guidance for practitioners as well, suggesting that the incorporation of hedging in advertisement messages may be particularly useful in persuading previous customers to use DTC products and services again. As previously noted, perhaps existing customers appreciate the perceived transparency afforded by the inclusion of hedging (e.g., “Although you should consult your medical provider before making serious decisions based on our genetic test results, our test provides helpful information to learn more about where you came from”), language that may be perceived as more helpful than promotional. Perhaps hedging as a message feature functions to maintain and reinforce consumer-brand relationships—an area for future research.

This study tested theoretical linkages informed by the theory of planned behavior and risk information seeking and processing, which are often tested using cross-sectional survey designs, in an experimental setting to observe potential causal relationships between variables. However, the study is not without limitations. Beyond limitations endemic to online experiments, the recruited sample was relatively homogenous with regard to race and ethnicity. Because of the study design and because data were self-reported from a single, relatively homogenous sample, the data may be prone to common method bias, discriminative bias, and a response carry-over effect [76–78]. Moreover, this study only examined how variables impacted purchase intentions; future research should examine how variables impact actual purchase behaviors and other meaningful behaviors, such as product recommendations to friends and family. Similarly, the stimuli shown to participants featured advertisements from a fictitious company designed to appear realistic. Examining variables of interest among actual or prospective customers of real DTC genetic testing companies would also advance theory and practice. Nonetheless, this study offers support for theory of planned behavior and risk information seeking and processing theoretical linkages in a DTC health product context and offers guidance for health communication practitioners and research moving forward.

## Supporting information

S1 File Study dataset. (CSV)

S1 Appendix (DOCX)
