# Supplementary material for: How message appeals and prior product use influence information processing, risk perceptions, trust, attitudes, and genetic test purchase intentions
Source: PLoS One. 2023 Mar 15;18(3):e0283102. doi: 10.1371/journal.pone.0283102 (PMC10016637; doi:10.1371/journal.pone.0283102)

**Appendix**

Condition 1: One-Sided/No Hedging


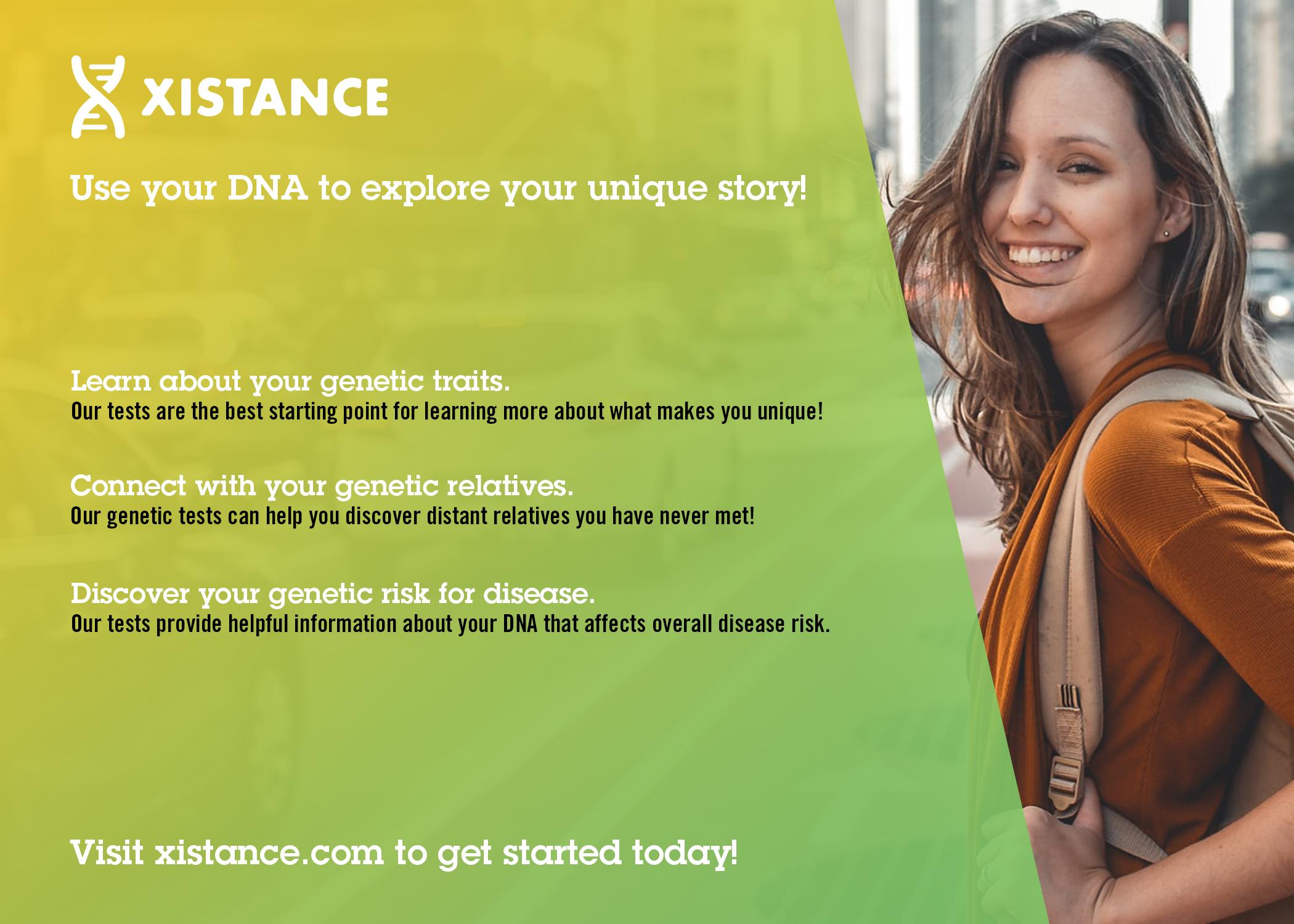


Condition 2: One-Sided/Hedging


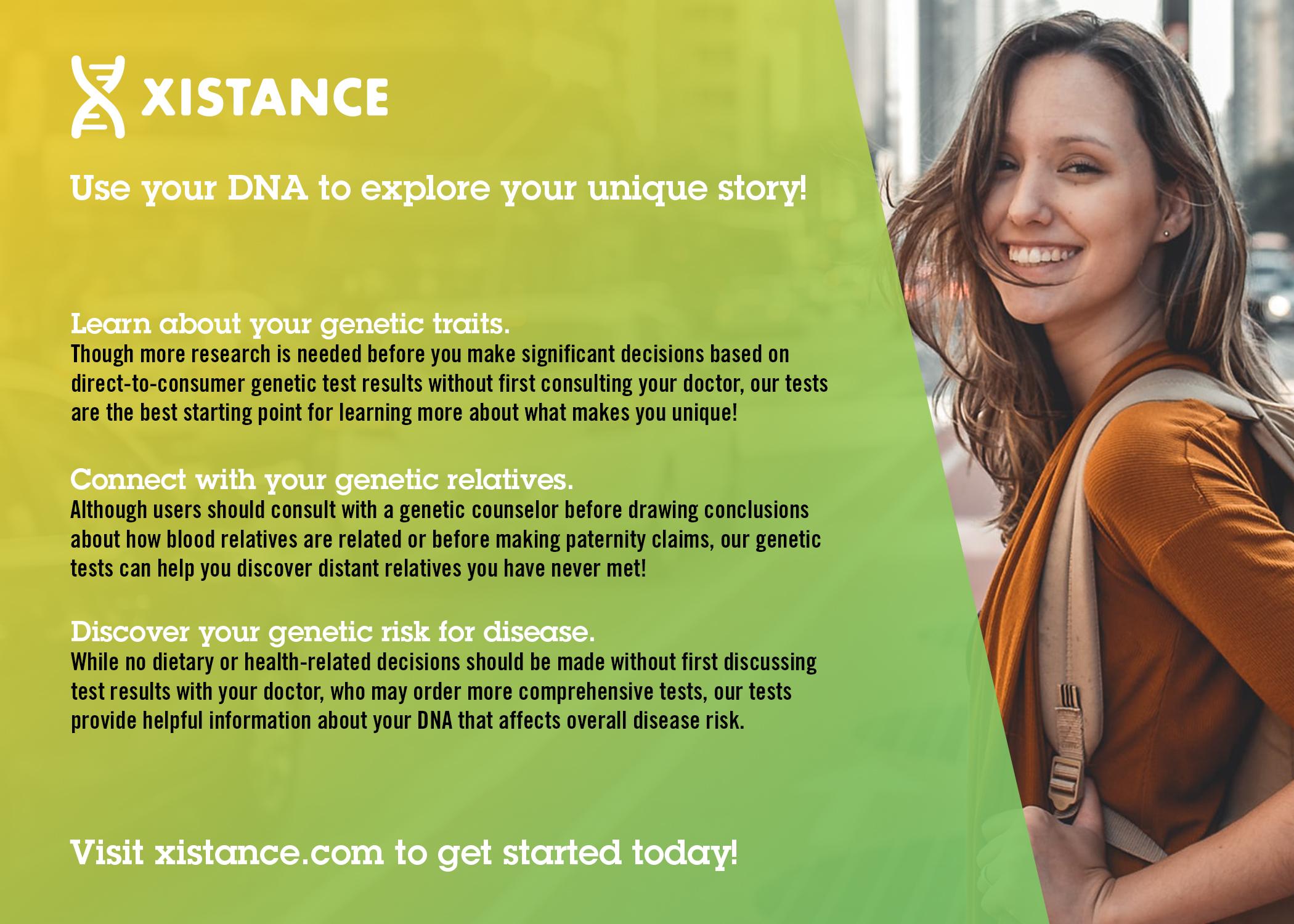


Condition 3: Two-Sided/No Hedging


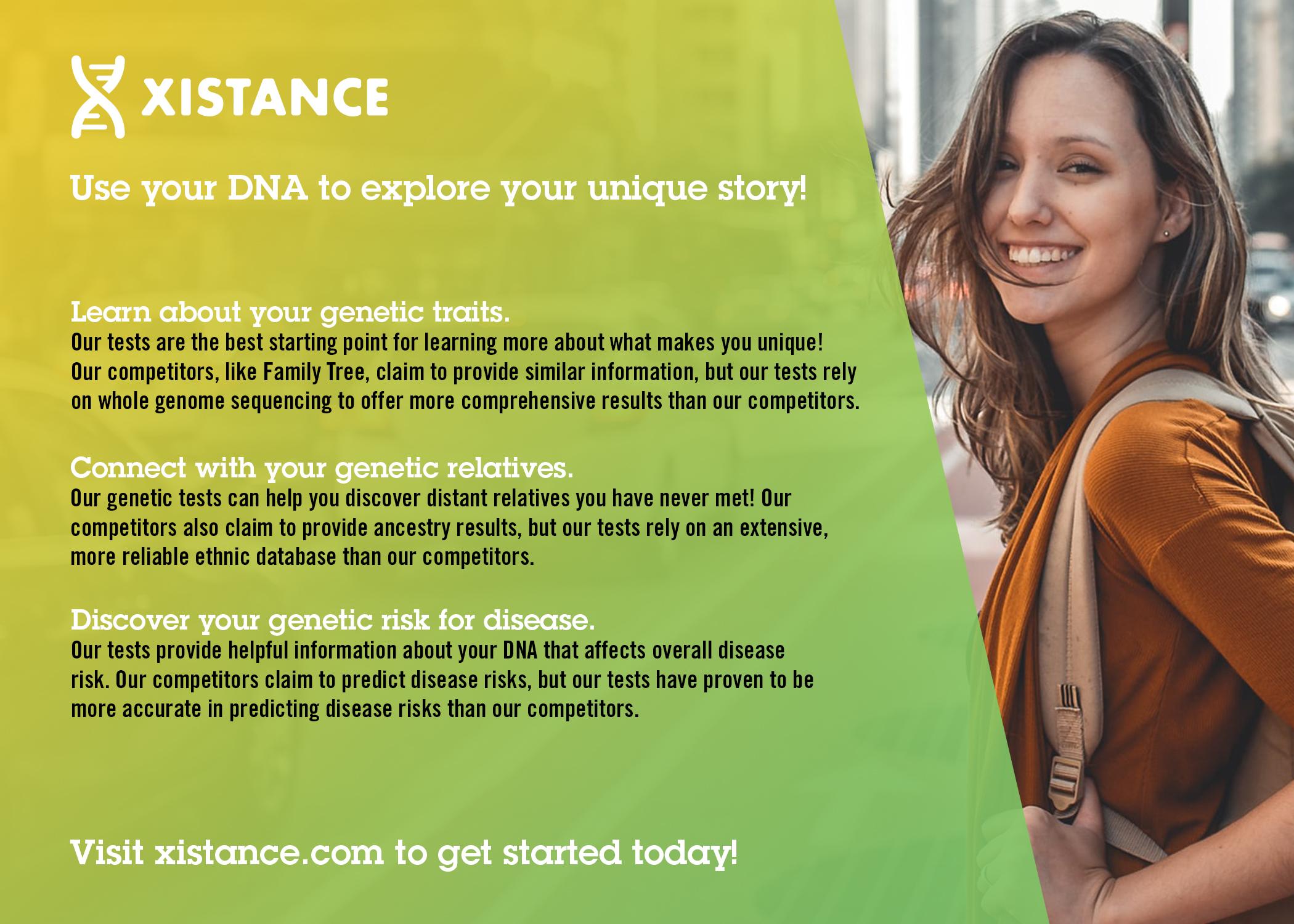


Condition 4: Two-Sided/Hedging


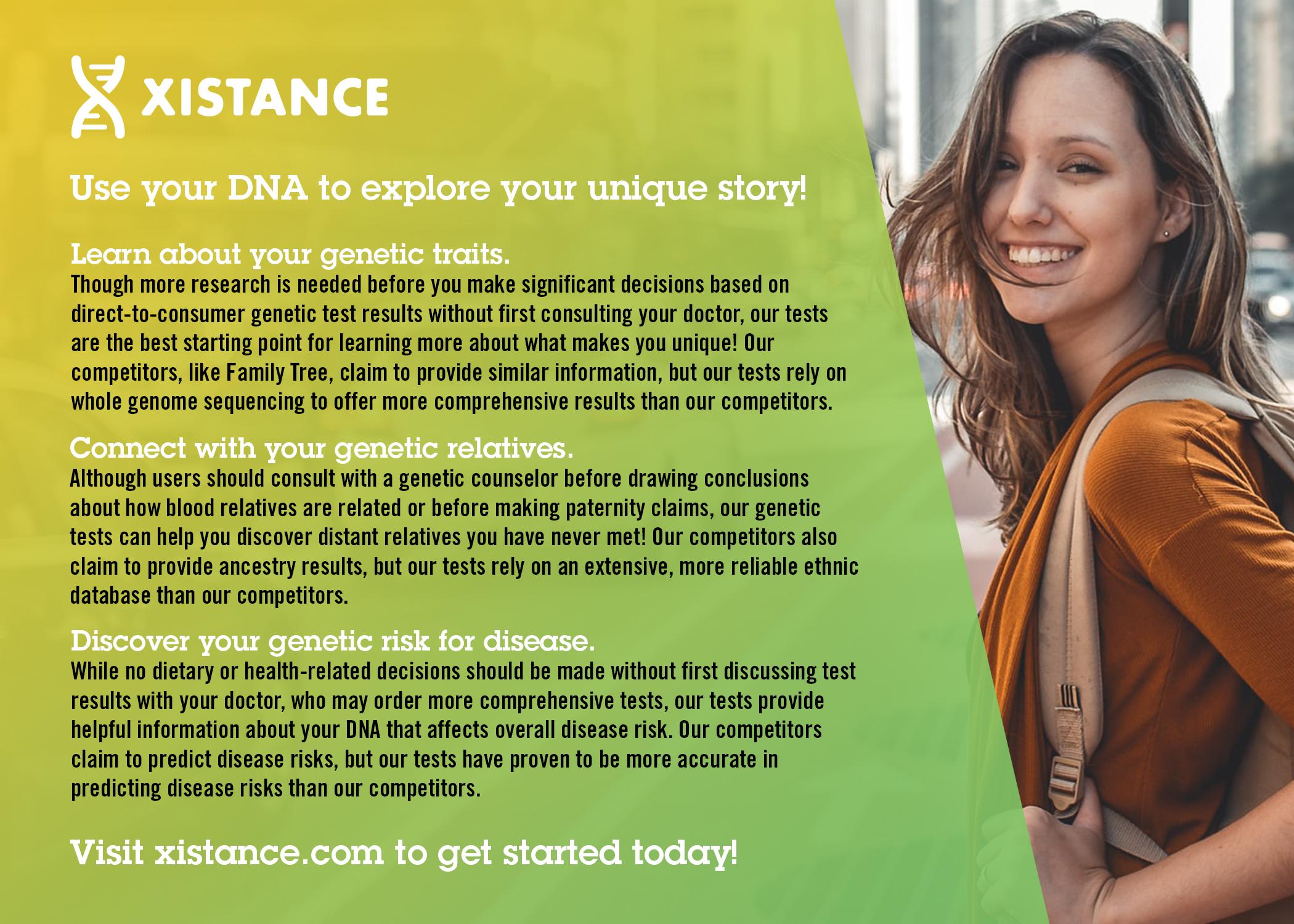

Supplement: S1 Appendix — (DOCX) [file pone.0283102.s002.docx]
